# A Longitudinal Analysis of Mortality Related to Chronic Viral Hepatitis and Hepatocellular Carcinoma in the United States

**DOI:** 10.3390/v16050694

**Published:** 2024-04-28

**Authors:** N. Begum Ozturk, Hoang Nhat Pham, Rama Mouhaffel, Ramzi Ibrahim, Marwan Alsaqa, Ahmet Gurakar, Behnam Saberi

**Affiliations:** 1 Department of Medicine, Beaumont Hospital, Royal Oak, MI 48073, USA; 2 Department of Medicine, University of Arizona Tucson, Tucson, AZ 85721, USA; 3 Division of Gastroenterology and Hepatology, Beth Israel Deaconess Medical Center, Harvard Medical School, Boston, MA 02130, USA; 4 Division of Gastroenterology and Hepatology, Johns Hopkins University School of Medicine, 720 Rutland Avenue, Ross Research Building, Suite 918, Baltimore, MD 21205, USA

**Keywords:** liver cancer, hepatitis B, hepatitis C, end-stage liver disease

## Abstract

(1) Background: Hepatocellular carcinoma (HCC) contributes to the significant burden of cancer mortality in the United States (US). Despite highly efficacious antivirals, chronic viral hepatitis (CVH) remains an important cause of HCC. With advancements in therapeutic modalities, along with the aging of the population, we aimed to assess the contribution of CVH in HCC-related mortality in the US between 1999–2020. (2) Methods: We queried all deaths related to CVH and HCC in the multiple-causes-of-death files from the CDC Wide-ranging Online Data for Epidemiologic Research (WONDER) database between 1999–2020. Using the direct method of standardization, we adjusted all mortality information for age and compared the age-adjusted mortality rates (AAMRs) across demographic populations and by percentile rankings of social vulnerability. Temporal shifts in mortality were quantified using log-linear regression models. (3) Results: A total of 35,030 deaths were identified between 1999–2020. The overall crude mortality increased from 0.27 in 1999 to 8.32 in 2016, followed by a slight reduction to 7.04 in 2020. The cumulative AAMR during the study period was 4.43 (95% CI, 4.39–4.48). Males (AAMR 7.70) had higher mortality rates compared to females (AAMR 1.44). Mortality was higher among Hispanic populations (AAMR 6.72) compared to non-Hispanic populations (AAMR 4.18). Higher mortality was observed in US counties categorized as the most socially vulnerable (AAMR 5.20) compared to counties that are the least socially vulnerable (AAMR 2.53), with social vulnerability accounting for 2.67 excess deaths per 1,000,000 person-years. (4) Conclusions: Our epidemiological analysis revealed an overall increase in CVH-related HCC mortality between 1999–2008, followed by a stagnation period until 2020. CVH-related HCC mortality disproportionately affected males, Hispanic populations, and Black/African American populations, Western US regions, and socially vulnerable counties. These insights can help aid in the development of strategies to target vulnerable patients, focus on preventive efforts, and allocate resources to decrease HCC-related mortality.

## 1. Introduction

Hepatocellular carcinoma (HCC) is the most common primary hepatic cancer and consists of approximately 90% of liver cancers [1]. HCC poses a global public health concern with its increasing incidence and currently is the third leading cause of cancer mortality [1,2]. Estimates suggest that HCC will affect approximately over 1 million individuals worldwide by 2025 [1,3]. In the United States (US), HCC is the fastest-growing cause of cancer and cancer-related mortality [4,5]. The most common risk factor for HCC is liver cirrhosis, which can be seen due to a variety of etiologies causing chronic liver disease and liver fibrosis progressing to cirrhosis, or in the setting of hepatitis B virus (HBV) infection, metabolic-dysfunction-associated steatatohepatis, diabetes mellitus, heavy alcohol use, the consumption of aflatoxin-containing products, or obesity without cirrhosis [1,6].

Chronic viral hepatitis (CVH), specifically those caused by HBV and hepatitis C virus (HCV) infections, remains the leading risk factor for cirrhosis and HCC development globally [7]. The mechanisms in which HBV and HCV cause carcinogenesis are complex and involve interactions between the virus and host. HBV is a DNA virus in the Hepadnaviridae family, and can lead to HCC through direct or indirect mechanisms including oxidative distress due to chronic viral infection and viral proteins, the alteration of the regulation of cell signaling pathways, and the integration of HBV DNA into the host genome [8,9]. HCV is an enveloped RNA virus in the Flaviviridae family, and promotes cellular proliferation; alters energy metabolism and apoptosis; causes chronic inflammation, steatosis, insulin resistance, oxidative distress, DNA damage, and genetic instability; and can lead to liver cirrhosis and HCC [1,10]. Unlike HBV, HCV cannot integrate into the host genome [7]. In patients with CVH, HCC may result from a cumulative effect of the combination of host (age, sex, genetic predisposition, alcohol use, diabetes mellitus, and metabolic syndrome), environmental (exposure to aflatoxins, organic solvents, and pesticides), and viral factors (co-infection with HBV, HCV, or HIV, HBV genotypes, HBV DNA level, positive hepatitis B e antigen, HBV mutations, HCV RNA level, and HCV genotype) [11,12,13,14].

In the US, approximately 3.5 million people are infected with HCV, and 1.59 million people are infected with HBV [15,16]. Despite highly successful antivirals for both HBV and HCV, and an effective vaccine for HBV, the 2015 Global Burden of Disease (GBD) reported HCV to be responsible for 31% and HBV for 9% as the major contributors to HCC-related mortality, with an increased HCC mortality projection until at least 2030 [6,17]. A comprehensive understanding of the CVH-related HCC mortality over the years is important given that highly successful antivirals exist for both HBV and HCV. In this study, we aimed to assess the mortality trends of HCC in the setting of CVH, with a focus on the impact of demographic factors and county-level social vulnerabilities in the US.

## 2. Methods

Data on the Social Vulnerability Index (SVI) and deaths related to HCC in patients with CVH within the US were obtained from the Centers for Disease Control (CDC)/Prevention Agency for Toxic Substances and Disease Registry (ATSDR) database for the 2020 release, and from the multiple cause of death files available through the CDC Wide-ranging Online Data for Epidemiologic Research (WONDER) database, respectively [18,19]. Mortality data were collected by querying using International Classification of Diseases, Tenth Revision (ICD-10) codes with all deaths related to CVH (ICD-10: B18.0, B18.1, B18.2, B18.8, and B18.9) and HCC (ICD-10: C22.0) in the multiple-causes-of-death records.

The SVI, developed and released by the CDC, is a measure of population-level characteristics of US counties’ potential resilience to multiple external stressors including pandemics, natural or human-caused disasters, and disease outbreaks. The SVI 2020 release comprises an aggregated 5-year estimate from the American Community Survey (2016–2020). Each US county’s overall SVI was obtained as overall percentile rankings from 0 to 1. Higher values indicated greater social vulnerability. County-level percentile rankings were grouped into four quartiles, ranging from first quartile Q1 (least socially vulnerable) to fourth quartile Q4 (most socially vulnerable). Excess or fewer deaths per 1,000,000 person-years were estimated between Q4 and Q1.

Crude mortality rates (CMRs) were calculated by dividing the number of deaths associated with the selected ICD-10 codes by the corresponding population size. Age-adjusted mortality rates (AAMRs) per 1,000,000 and 95% confidence intervals were estimated using a direct method of standardization to the US population in the year 2000. AAMR comparative analyses across all subpopulations were performed based on demographic factors, including sex, race and ethnicity, urbanization, US census region, and SVI quartiles. Log-linear regression models (Joinpoint Regression; National Cancer Institute) were used to analyze AAMR temporal variations by identifying inflection points in mortality trends [20]. Monte Carlo permutation test was used to estimate the annual percentage change (APC) and weighted averages of the APC, yielding average annual percentage change (AAPC). Statistical analysis was completed by using a two-tailed t-test to assess significant shifts in APC, with a significance threshold set at *p* < 0.05. Approval from an Institutional Review Board was not required since the de-identified and publicly available data were gathered by the CDC for surveillance purposes.

## 3. Results

**Figure 1:**
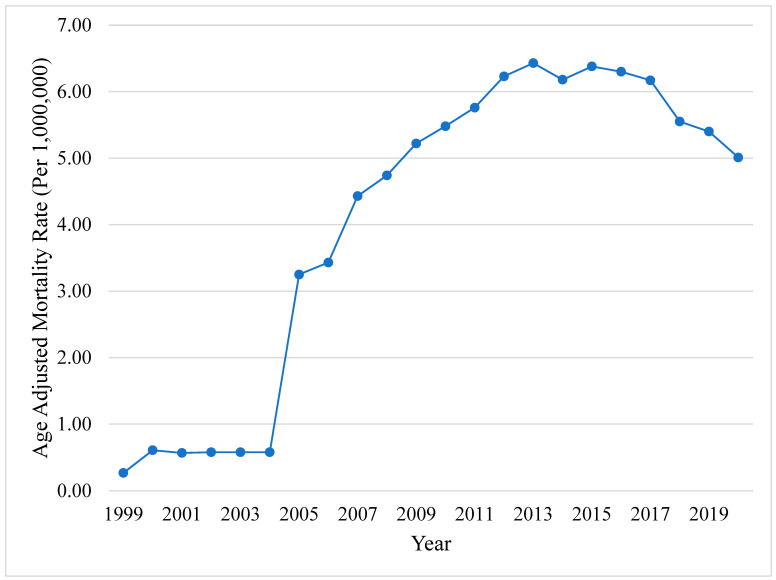
**Hepatocellular carcinoma in patients with chronic viral hepatitis.** Yearly AAMR in the US from 1999 to 2020. Abbreviations: AAMR = age-adjusted mortality rate, US = United States.

A total of 35,030 HCC and CVH deaths were identified between 1999 and 2020. The overall CMR increased from 0.27 [95% CI, 0.21–0.33] in 1999 to 7.04 [95% CI, 6.76–7.33] in 2020. Similarly, the AAMR demonstrated a comparable trend, rising from 0.27 [95% CI, 0.21–0.34] in 1999 to 5.01 [95% CI, 4.81–5.22] in 2020, with an AAPC of 15.15 [95% CI, 10.63–19.85, *p* < 0.001] (Figure 1). The mortality rate increased from 1999 to 2008 (APC +39.31, *p* < 0.001) with a stalling in mortality from 2008 to 2020 (APC −0.18, *p* = 0.88). The cumulative AAMR during our 22-year study period was 4.43 (95% CI, 4.39–4.48).

**Figure 2:**
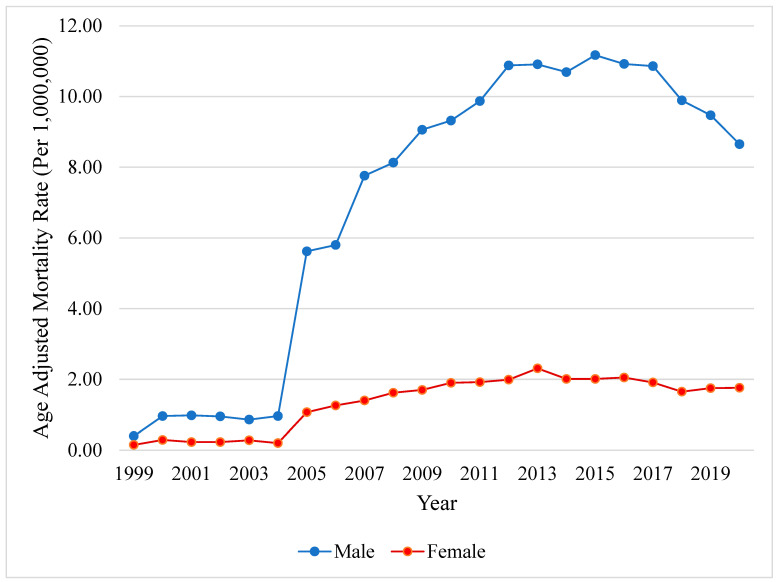
**Hepatocellular carcinoma in patients with chronic viral hepatitis, stratified by sex**. Yearly AAMR in the US from 1999 to 2020. Abbreviations: AAMR = age-adjusted mortality rate, US = United States.

Male decedents (AAMR 7.70 [95% CI, 7.61–7.79]) were impacted by higher mortality rates compared to female decedents (AAMR 1.44 [95% CI, 1.40–1.48]). Mortality increased sharply in male decedents from 1999 to 2007 (APC +44.89, *p* < 0.001), followed by two decelerating inflection points in 2007 (APC +4.31, *p* = 0.10) and in 2015 (APC −5.19, *p* = 0.17). Mortality among female decedents remained consistent from 1999 to 2003 (APC +9.90, *p* = 0.33), followed by a surge between 2003 and 2006 (APC +70.19, *p* = 0.04) and two decelerating points in 2006 (APC +6.69, *p* = 0.001) and in 2013 (APC −3.81, *p* = 0.004) (Figure 2).

**Figure 3:**
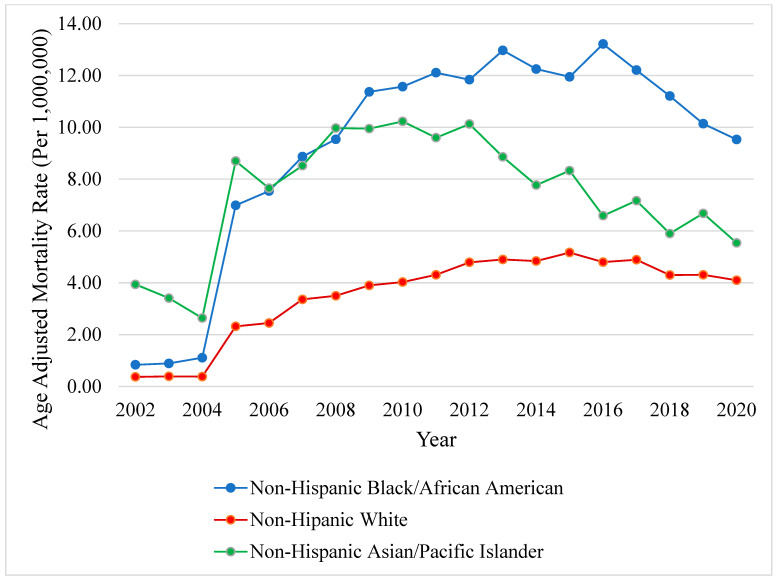
**Hepatocellular carcinoma in patients with chronic viral hepatitis, stratified by race**. Yearly AAMR in the US from 1999 to 2020. Abbreviations: AAMR = age-adjusted mortality rate, US = United States.

Mortality was higher among Hispanic decedents (AAMR 6.72 [95% CI, 6.53–6.92]) compared to non-Hispanic decedents (AAMR 4.18 [95% CI, 4.14–4.23]). Among the non-Hispanic decedents, mortality was highest among Black/African American decedents (AAMR 9.06 [95% CI, 8.85–9.27]), followed by American Indian/Alaska Native (AAMR 7.56 [95% CI, 6.82–8.30]), Asian/Pacific Islander (AAMR 7.25 [95% CI, 6.94–7.55]), and White (AAMR 3.33 [95% CI, 3.29–3.38]) decedents. Mortality among Black decedents increased significantly from 2002 to 2005 (APC +144.40, *p* < 0.001), followed by a decelerating period between 2005 and 2011 (APC +11.72, *p* = 0.001), and decreasing mortality after 2011 (APC −2.48, *p* = 0.02). Similar patterns were observed in White decedents with an uptrend in mortality between 2002–2005 (APC +121.54, *p* < 0.001), followed by a decelerating inflection point starting in 2005 until 2012 (APC +11.53, *p* < 0.001), followed by a mortality decline in 2013 (APC −2.17, *p* = 0.02). In contrast, Asian/Pacific Islander decedents showed different mortality trends, with an upward trend between 2002–2008 (APC +21.32, *p* = 0.001) and a decreasing trend between 2008–2020 (APC −5.14, *p* < 0.001) (Figure 3).

**Figure 4:**
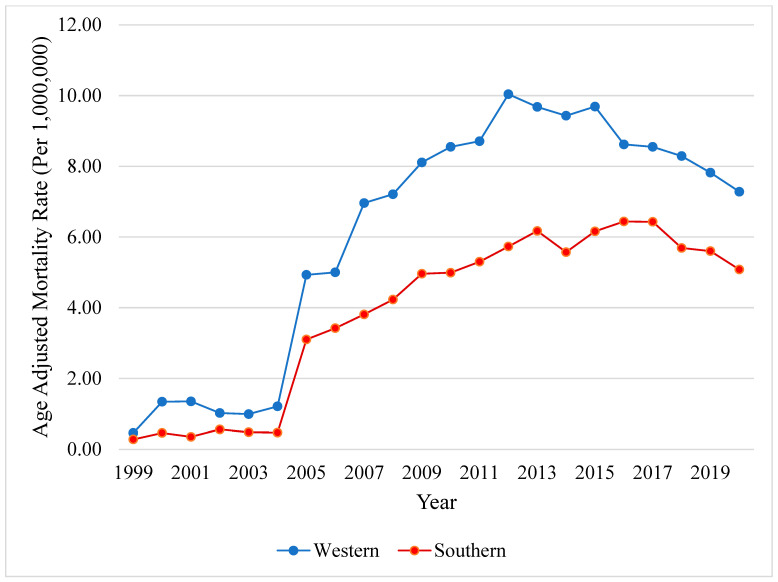
**Hepatocellular carcinoma in patients with chronic viral hepatitis, stratified by US census region**. Yearly AAMR in the US from 1999 to 2020. Abbreviations: AAMR = age-adjusted mortality rate, US = United States.

Mortality was higher among metropolitan regions (AAMR 4.64 [95% CI, 4.58–4.69]) compared to non-metropolitan regions (AAMR 3.42 [95% CI, 3.32–3.52]). Mortality was highest among the Western (AAMR 6.75 [95% CI, 6.62–6.87]), followed by Southern (AAMR 4.27 [95% CI, 4.20–4.35]), Northeastern (AAMR 3.34 [95% CI, 3.25–3.44]), and Midwestern US regions (AAMR 3.30 [95% CI, 3.21–3.38]). Mortality increased sharply in the Western US region between 1999–2007 (APC +35.80, *p* < 0.001), followed by mortality stagnation between 2007–2013 (APC +6.36, *p* = 0.14), and a decreasing mortality trend after 2013 (APC −4.28, *p* = 0.06). Similarly, the mortality rate in Southern states increased between 1999–2006 (APC +54.95, *p* < 0.001), followed by a decelerating inflection point starting in 2006 until 2016 (APC +5.63, *p* = 0.005), and mortality stagnation starting in 2016 until 2020 (APC −6.5, *p* = 0.19) (Figure 4).

Higher CVH-related HCC mortality was observed in US counties categorized as Q4 (AAMR 5.20 [95% CI, 5.11–5.29]) than Q1 (AAMR 2.53 [95% CI, 2.43–2.62]). These higher levels of social vulnerability were associated with an excess of 2.67 deaths per 1,000,000 person-years. Similar trends were observed when stratified by sex, ethnicity, urbanization, and US census regions (Table 1).

## 4. Discussion

Our study evaluated the CVH-related HCC mortality trends during a time period that coincided with the introduction of antiviral therapy for HBV and HCV in the late 2000s and early 2010s, respectively, in the US [7,21,22]. Despite the initial interferon-based HCV treatment approved in 1997 showing limited success and a high rate of adverse effects, the introduction of interferon-free direct-acting antiviral (DAA) therapy in 2012 marked a significant advancement in the management of HCV [23]. Combination DAA therapies have achieved cure rates exceeding 95% with a sustained virologic response (SVR), which has been associated with decreased liver-related outcomes including HCC and mortality [21]. However, the risk of HCC may persist even after a successful SVR if advanced fibrosis is present [21,24]. Similarly, highly effective antivirals for HBV were introduced, with entecavir receiving FDA approval in 2005, and tenofovir disoproxil fumarate in 2008, both demonstrating high rates of the durable virologic response [7]. These medical advancements may have contributed to the stagnation observed in the CVH-related HCC mortality trends from the late 2000s, preceded by a significant increase between 1999 and 2008 in our study. Our mortality analysis was similar to a previous study, which reported that HCV-related HCC deaths increased from 1999 to 2012, followed by a subsequent declining trend until 2018 in the US [25]. Another study, utilizing the Surveillance, Epidemiology, and End Results (SEER)–Medicare data to assess the HBV and HCV status in patients with HCC aged over 66 years of age demonstrated a substantial increase in HCV-attributable HCC cases during 2001–2013, with a slower increase in HBV-attributable HCC cases in the US [26].

Our results unveiled a disproportionate distribution of CVH-related HCC deaths between males and females, consistent with prior epidemiological studies on HCC-related mortality in the general population [27,28]. The higher prevalence of liver-related comorbidities in males, including alcohol-associated liver disease, metabolic-dysfunction-associated steatotic liver disease, metabolic syndrome, and tobacco use, could exacerbate the progression of CVH and increase the risk of HCC in these demographic [29,30]. Other factors contributing to this sex-based disparity may include delayed diagnosis, treatment initiation, and medication compliance among males compared to females [31].

HCC exhibits an unequal distribution by race/ethnicity, with older Black/African American patients displaying significantly higher incidence and mortality rates of HCC compared to White patients [27]. The overall mortality attributed to HCC among Black/African American patients, in excess relative to White patients, increased from 27.8% to 45.5% between 1998–2016 [32]. In our study, when stratified by race/ethnicity, Black/African American decedents exhibited the highest AAMR starting from 2008, while before 2008, Asian/Pacific Islander decedents had the highest AAMR. In the US, approximately half of the patients with HBV are of Asian/Pacific Islander descent, and individuals who were born outside of the US and migrated later constitute over 70% of the HBV cases, and the treatment with nucleotide/nucleoside analogs, which mitigates the risk of HCC, is thought to be responsible for the observed decline in mortality rates in this population beginning in the mid-2000s [33,34,35,36]. Furthermore, the decreased rates of HCC-related mortality may also be attributed to the increased vaccination and treatment of HBV, and the availability of DAAs against HCV beginning in late 2014 [34,37]. The sharp increase in CVH-related HCC mortality around 2005 may be explained by the increased rates of diagnosis of CVH and HCC in the USA, in particular, for the years 2003–2005, the increased incidence and mortality in the Asian/Pacific Islander population and Black/African American men as reported by one study [38]. Similarly, another recent study also reported the increased incidence of all causes of HCC, in particular, in non-Hispanic White men until 2006, and with a peak incidence of HCC in non-Hispanic American Indian or Alaskan Native men aged <55 in 2006 [39]. The same study also reported an increased mortality trend between 2004–2006 in non-Hispanic Black/African American patients aged <55, similar to our study [39]. Without effective linkage through the proactive identification and detection of HBV, in particular in high-risk populations, mortality rates may not improve despite highly successful vaccination and antiviral therapies.

Overall, all US racial/ethnic populations with CVH have experienced a declining trend of HCC-related AAMRs in recent years, similar to a prior study conducted from the SEER database which reported a decrease in HCC mortality rates across most populations from 2015 onwards [40]. However, the onset of this decline varied among these populations in our study, an indication of potential disparities that persists. These disparities between race/ethnicity are further influenced by socioeconomic disadvantages and discrepancies in healthcare access [34,41]. Previous studies reported that Black/African American patients with CVH are less likely to receive treatment compared to White patients [42]. Additionally, they tend to have more advanced tumor stages and are more likely to have metastasis at the time of initial HCC diagnosis, and are less likely to undergo local or surgical curative therapy, even when the tumor is localized to the liver, and are less likely to receive liver transplantation with worse post-liver transplant outcomes compared to other racial/ethnic populations [43,44,45]. In a study conducted by using the National Inpatient Sample involving 62,604 patients with all causes of HCC, Black/African American individuals had similar disparities in the rates of curative treatment with resection and ablation, and a worse disease stage at the time of HCC diagnosis, and are less likely to receive curative therapy or liver transplantation in the Southern US [45].

Although many studies have documented higher cancer-related mortality in rural settings compared to urban settings, HCC differs from other cancers as its at-risk population predominantly comprises individuals with cirrhosis that necessitates distinct surveillance measures [46,47]. Our results indicated a higher HCC-related mortality in CVH in metropolitan areas compared to rural areas among SVI-Q2, SVI-Q3, and SVI-Q4 populations. Several studies have reported higher mortality rates in rural areas compared to urban areas for HCC across all etiologies [46,48]. However, a study also noted race to be a stronger predictor of overall survival than rural residency in patients with HCC [49]. While these disparities are not exclusive to any particular racial or ethnic group, they are influenced by a multitude of intricate behavioral and sociocultural factors [41].

Our study has several limitations. We queried death certificate information to obtain aggregated decedent information; thus, individual-level patient data were not accessible. Consequently, we were unable to adjust for factors such as the comorbidity burden, other confounders for liver disease (e.g., obesity, metabolic dysfunction, and alcohol use), the specific type of CVH, or history of a treatment for CVH. Furthermore, relying on death certificates makes our results susceptible to errors in ICD-10 coding. As the CDC has a policy of suppressing annual death rates due to low death counts, certain racial and ethnic groups such as the Hispanic population was precluded from a detailed temporal mortality analysis. In addition, our data are not generalizable to other countries as significant disparities exist for the epidemiology of CVH-related HCC in the world between high-income countries such as the US, and low-income countries [50]. Lastly, given the use of population-level data, the ecological fallacy remains a hindrance to applying this epidemiological knowledge to the patient level. However, these findings are hypothesis-generating and warrant further investigation into these disparities.

## 5. Conclusions

HCC remains a major public health issue, disproportionately affecting minority populations and metropolitan US regions. Although multiple risk factors could also contribute to the mortality in patients with CVH-related HCC, identifying and linking patients to treatment for viral eradication remains important. Although the limitations of our analysis warrant cautious interpretation, these findings provide a valuable understanding of contemporary CVH-related HCC mortality disparities, supporting the development of targeted interventions.

## Figures and Tables

**Table 1:** Hepatocellular carcinoma related age-adjusted mortality in population with chronic viral hepatitis among social vulnerability quartiles.

	SVI-Q1 [95% CI]	SVI-Q2 [95% CI]	SVI-Q3 [95% CI]	SVI-Q4 [95% CI]
All	2.53 [2.43–2.62]	3.82 [3.74–3.91]	4.99 [4.90–5.08]	5.20 [5.11–5.29]
Sex				
Female	0.80 [0.73–0.88]	1.24 [1.17–1.30]	1.64 [1.56–1.71]	1.72 [1.65–1.79]
Male	4.36 [4.18–4.53]	6.62 [6.45–6.78]	8.67 [8.49–8.85]	9.15 [8.98–9.33]
Ethnicity				
Hispanic	4.66 [3.73–5.59]	6.77 [6.17–7.37]	8.66 [8.17–9.14]	6.22 [5.98–6.45]
NH	2.49 [2.40–2.58]	3.70 [3.61–3.79]	4.70 [4.61–4.80]	4.95 [4.85–5.04]
Race				
NH Black	8.38 [7.36–9.40]	9.99 [9.40–10.59]	11.11 [10.65–11.58]	7.99 [7.73–8.25]
NH White	2.25 [2.15–2.34]	2.99 [2.91–3.08]	3.81 [3.71–3.90]	3.93 [3.83–4.04]
NH Asian	5.53 [4.43–6.62]	8.46 [7.81–9.11]	7.92 [7.35–8.50]	6.20 [5.74–6.66]
NH Native	NA	9.49 [7.60–11.71]	9.87 [8.16–11.58]	6.21 [5.26–7.16]
Census Region				
Northeast	2.65 [2.45–2.84]	3.01 [2.86–3.17]	3.22 [3.05–3.40]	4.56 [4.33–4.80]
Midwest	2.17 [2.04–2.29]	3.07 [2.91–3.23]	4.60 [4.39–4.81]	4.02 [3.78–4.26]
South	2.57 [2.33–2.82]	3.15 [3.01–3.29]	4.43 [4.29–4.57]	4.90 [4.78–5.02]
West	4.12 [3.72–4.51]	6.99 [6.72–7.27]	7.37 [7.15–7.59]	6.51 [6.32–6.71]
Urbanization				
Urban	2.59 [2.48–2.70]	4.01 [3.92–4.11]	5.26 [5.15–5.36]	5.36 [5.26–5.46]
Rural	2.34 [2.14–2.53]	2.96 [2.77–3.15]	3.61 [3.41–3.81]	4.36 [4.15–4.57]

Abbreviations: NA = not applicable, NH = non-Hispanic, Q = quartile, SVI = social vulnerability index.

## Data Availability

Public data is available through CDC-WONDER database.
